# Routine closed-suction drainage reduces seromas following totally extraperitoneal (TEP) inguinal hernia repair: A meta-analysis

**DOI:** 10.1097/MD.0000000000037412

**Published:** 2024-03-15

**Authors:** Dimitrios Prassas, Michael Zaczek, Stephan Oliver David, Wolfram Trudo Knoefel, Sascha Vaghiri

**Affiliations:** a Katholisches Klinikum Essen, Department of Surgery, Essen Germany; b Heinrich-Heine-University and University Hospital Duesseldorf, Department of Surgery (A), Duesseldorf, Germany.

**Keywords:** inguinal hernia, meta-analysis, seroma, TEP, total extraperitoneal

## Abstract

**Background:**

The value of prophylactic closed-suction drainage in totally extraperitoneal inguinal hernia repair (TEP) is still a matter of controversy. We conducted a meta-analysis of studies examining postoperative seroma rates in patients with or without routine placement of closed-suction drainage tubes.

**Methods:**

A systematic literature search was conducted for trials comparing the outcome of TEP with or without routine drainage placement. Data regarding postoperative outcomes were extracted and compared by meta-analysis. The odds ratio and standardized mean differences with 95% confidence intervals were calculated.

**Results:**

Four studies were identified, involving a total of 1626 cases (Drain: n = 1251, no Drain: n = 375). There was a statistically significant difference noted between the 2 groups regarding postoperative seroma formation favoring the Drain group (odds ratio = 0.12; 95% confidence intervals [0.05, 0.29]; *P* < .001; 4 studies; *I*^2^ = 72%). For the remaining secondary endpoints postoperative urinary retention, recurrence, mesh infection and in-hospital length of stay no statistically significant difference was noted between the 2 study groups.

**Conclusion:**

Current evidence suggests that patients who underwent TEP with routine closed-suction drain placement developed significantly fewer seromas without any additional morbidity or prolongation of in-hospital stay.

## 1. Introduction

Every day thousands of patients worldwide undergo inguinal hernia repair. In Germany alone about 200,000 cases are operated annually.^[1]^ The minimally invasive approach has been proven to be an efficient and safe method providing excellent operative outcomes.^[2,3]^ Nevertheless, postoperative seroma formation remains a relatively frequent sequela after totally extraperitoneal hernia (TEP) repair in the early postoperative period^[4]^ with prevalence that reaches 37.9% in the first week after discharge.^[5]^ Most of the cases resolve spontaneously but others require sterile aspiration. Considering the dimensions of the mycopectineal space that is being dissected in TEP surgery, it can be said that after surgery, the extraperitoneal space is no longer our working space but a dead one. Adhesions are formed rapidly, being accelerated by the presence of the obligatory prosthetic material, but the early postoperative phase is the one where the seroma can occur and establish its presence in the recently dissected space. As a result, it has been hypothesized that the routine placement of closed-suction drains can eliminate the dead space in the early postoperative period and thus prevent seroma formation.^[6–9]^

The aim of this study was to perform a meta-analysis of studies comparing the feasibility and safety routine closed-suction drainage placement after TEP for inguinal hernia repair in terms of postoperative seroma formation.

## 2. Materials and methods

This systematic meta-analysis was conducted according to the “Preferred Reporting Items for Systematic Reviews and Meta-analyses” statement.^[10]^

### 2.1. Eligibility criteria

All studies comparing the outcome of TEP in cases conducted with routine closed-suction drainage versus cases without drainage were considered for inclusion, regardless of study size. To be included in the analysis, studies had to report on at least the rate of postoperative seroma.

### 2.2. Search strategy

A systematic review was independently conducted by 2 authors (DP and SV) in MEDLINE, and the Cochrane CENTRAL trials register. No language restrictions were applied. Selected papers were screened by both reviewers for eligibility. Discrepancies that arose were resolved by consensus. If needed, a third author (MZ) was consulted. The last search was performed on 17.11.2023. The combination of the following medical subject headings was used to perform the search: ((tep OR “total extraperitoneal” OR “endoscopic hernia repair”) AND (drain*)) AND ((inguinal OR groin) OR seroma)).

### 2.3. Data extraction and outcome measures

A self-designed data extraction form was utilized to independently and blindly extract data of interest in included papers. Primary outcome of interest was postoperative seroma. Ismail et al^[6]^ defined seroma as “a non-tender hemispherical swelling with a fluctuant or firm consistency at the hernia site.” A similar definition has been given by Gao et al^[9]^ as “localized hemispherical swelling without tenderness, at the hernia site.” Fan et al^[7]^ defined seroma as “fluid collection with no doppler signal at the preperitoneal space on ultrasound examination or clinically palpable irreducible swelling after TEP hernioplasty without cough impulse” whereas Wu et al^[8]^ implemented a more general definition of seroma as “the exudation and accumulation of fluid at the operation area.” Secondary outcomes of interest included postoperative urinary retention, hernia recurrence and length of hospital stay. Recorded baseline study characteristics included year of publication, chronic pain, study type, study origin, study duration, sample size, age, gender, number of surgeons involved and surgical skill level, type of implanted mesh, mesh fixation, type of fixation, type of closed-suction drain used, time point of drainage removal, type of energy source used, type of follow-up, and duration of follow-up.

### 2.4. Quality assessment

The risk of bias of included studies was assessed independently by 2 authors (S.O.D. and M.Z.) using the Robins-I tool^[11]^ for non-randomized studies and the Rob2 criteria^[12]^ for randomized trials, respectively. In summary, the Robins-I tool evaluates the risk of bias ranging from low to critical in non-randomized studies based on 7 different bias domains (confounding, selection of participants, classification of interventions, deviations from intended interventions, missing data, measurement of outcomes, selection of reported results). Concomitantly, Rob2 criteria were able to classify randomized studies with respect to critical assessment of 5 potential bias domains into low to high risk of bias. The level of evidence for the significant outcomes was classified into 4 categories (high, moderate, low, and very low) according to GRADE (The Grading of Recommendations, Assessment, Development, and Evaluation).^[13]^

Disagreements in grading were discussed and resolved by consensus or reassessment by a third author (S.V.). The methodological quality of the present meta-analysis was ranked as `high` after implementation of AMSTAR 2 appraisal tool for systematic reviews that include randomized or non-randomized studies of healthcare interventions.^[14]^

### 2.5. Statistical analysis

Data of interest was analyzed with pairwise meta-analyses. For each outcome of interest summary estimates of treatment effect were calculated with 95% confidence interval (CI). The odds ratio (OR) was chosen as an effect measure for dichotomous endpoints. Standardized mean differences (SMD) were calculated to analyze continuous outcomes. The amount of variation by heterogeneity was assessed by the *I*^2^ index. Values exceeding 50% were regarded as markers of substantial heterogeneity. *I*^2^ values above 75% were regarded as markers of high heterogeneity. Summary estimates were calculated with a fixed-effects method in case of low or moderate heterogeneity (*I*^2^ < 50%). All meta-analyses were conducted with the RevMan software (Version 5.3. Copenhagen: The Nordic Cochrane Centre, The Cochrane Collaboration, 2014).

## 3. Results

### 3.1. Study selection and characteristics

**Figure 1.**
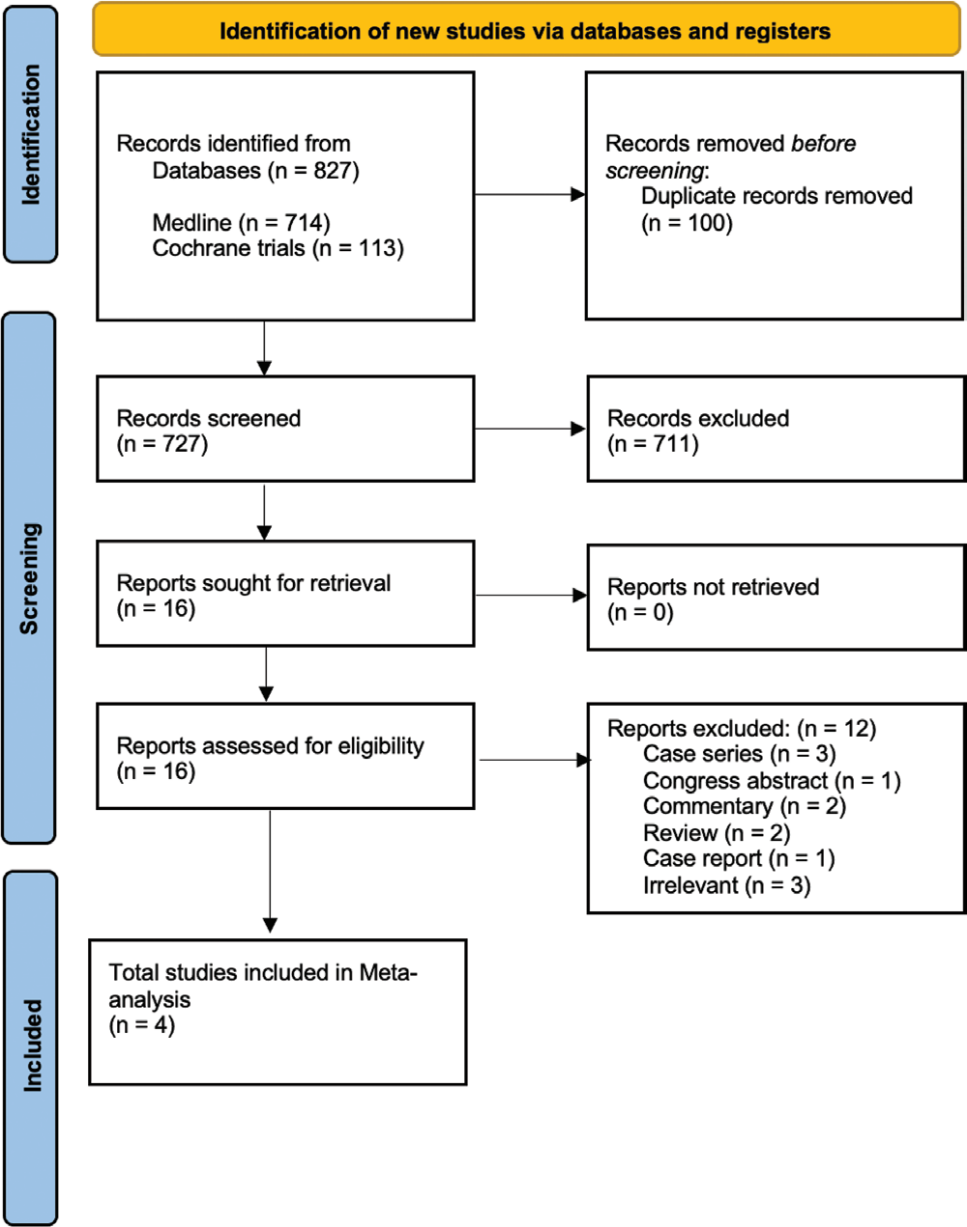
Flow chart.

Using our predefined literature search strategy and as shown by the PRISMA flow chart, electronic database search identified 727 studies, excluding 100 duplicates (Fig. 1). Sixteen full-text manuscripts were screened, 12 of which were excluded for different reasons (Fig.1). Overall, 4 studies were included in the qualitative and quantitative data synthesis, involving a total of 1626 cases (Drain: n = 1251, no Drain: n = 375).

Three studies were comparative retrospective cohorts.^[6,8,9]^ One study was a RCT.^[7]^ The inserted mesh was routinely fixated in one study^[7]^ either with nonabsorbable tacks or with glue. In the study of Ismail et al^[6]^ the mesh was not routinely fixated. In the study of Wu et al^[8]^ and Gao et al^[9]^ no mesh fixation took place. Three out of 4 studies report the diameter of the inserted drainage tube. Fan et al^[7]^ used an 8 Fr. tube whereas Ismail et al^[6]^ and Gao et al^[9]^ preferred a larger 12 Fr. tube. In all studies the tube was removed in the first 48 hours after surgery. Table 1 provides an overview of the study characteristics.

**Table 1:** Study characteristics.

Study	Fan 2017		Ismail 2009			Wu 2023				Gao 2015		
Origin	Hong Kong		India			China				China		
Time period	May 16–February 17		January 05–December 07			June 18–June 21				March 09–March 14		
Patient group	Drain	No Drain	Drain		No Drain	Drain			No Drain	Drain		No Drain
Patients	41	37	849		80	40			60	321		157
Age	53.5 ± 14.7	48.9 ± 18.7	46.8 ± 13.8		39.0 ± 15.8	60.7 ± 15.4			63.3 ± 13.9	54.2 ± 23.8		47.6 ± 26.8
Sex (M:F)	39:2	35:2	840:9		79:1	39:1			57:3	275:46		122:35
Number of surgeons	n/a		n/a			n/a				n/a		
Surgical skill level	“Hernia specialists or supervised trainees”		n/a			n/a				n/a		
Drains	8 Fr. Silicone		12 Fr.			‘Standard closed suction drainage tube’				12 Fr.		
Drainage removal	23 hours postop		24 hours postop			48h postop				48 hours postop.		
Operative time (minutes)	91.1 ± 35.9	96.9 ± 32	30.43 ± 6.0		28.7 ± 6.8	43.3 ± 15.2			40.8 ± 13.5	39 ± 9.4		35 ± 8.1
Hernia size	2.68 ± 1.13 direct 2.23 ± 0.68 indir.	2.21 ± 1.11 direct 2.11 ± 0.6 indir.	n/a		n/a	n/a			n/a	Scrotal: 42		Scrotal: 25
Bilateral hernias (n)	0		758		66	0				28		
Energy source	Monopolar electric		Bipolar electric			Bipolar electric				n/a		
Mesh fixation	Ti Tacks: n = 28 glue: n = 13		Tacks: n = 28		Tacks: n = 3	No fixation				No fixation		
Mesh type	Parietex anatomical^TM^		10 × 15 cm Polypropylene			`a prosthetic mesh’				n/a		
Exclusion criteria	Irreducible, recurrent, bilateral		Strangulated hernias			Bilateral, irreducible recurrent				n/a		
Follow Up length in months	7	7.6	Median: 22 (9–45)			3				Median: 24 (2–41)		
Follow Up rate	40/41		100%			100%				100%		
Follow Up Type	Clinical + sonography		Clinical			?				Clinical		
Intraoperative morbidity	0		0			0				0		
Conversion to open surgery	0		1	0			0			n/a		
Seroma	17/41	30/37	12/1607 hernias	22/146 hernias			2/40	12/60			13/321	29/157
Urinary retention	2/41	0	36	14			1/40	9/60			n/a	
Recurrence	0		2/1607	0			N/A			2/321		1/157
In-hospital stay in days	2.134 ± 0.738	2.204 ± 0.625	1.079 ± 0.28	1.017 ± 0.13			2.1 ± 0.9	2.0 ± 0.8			2.96 ± 0.35	3.05 ± 0.31

### 3.2. Study quality and risk of bias

**Figure 2.**
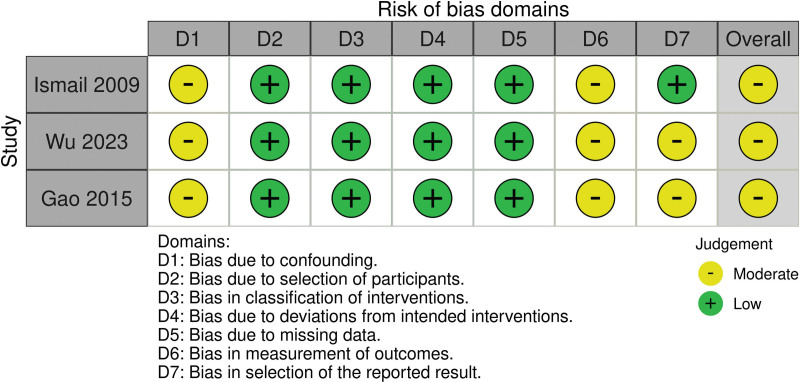
Potential sources of Bias—Robins 1.

**Figure 3.**
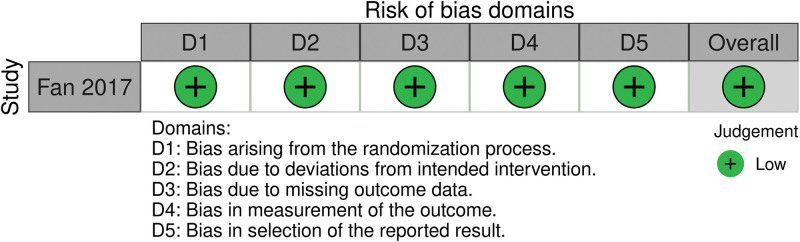
Potential sources of Bias—RoB2.

Potential sources of bias are summarized in Figures 2 and 3. According to Rob2, the risk of bias in the only included randomized study was low while the overall risk of bias in all non-randomized studies was moderate applying the ROBINS-I tool.

The main limitations arise from the fact that in surgical intervention trials, the technique used is always evident to the operating surgeon and most of the times evident to the patient as well, rendering blinding of patients and outcome assessors practically impossible. The retrospective design of 3 out of 4 trials^[6,8,9]^ also constitutes a significant risk of bias.

Furthermore, the inclusion of bilateral hernias in 2 studies^[6,9]^, large scrotal and irreducible hernias in one study^[9]^, and the wide time span of follow-up examination ranging from only 3 months in the study of Wu et al^[8]^ up to 45 months reported by Ismail et al^[6]^ may influence comparability of the presented results.

### 3.3. Primary outcome

#### 3.3.1. Postoperative seroma.

**Figure 4.**
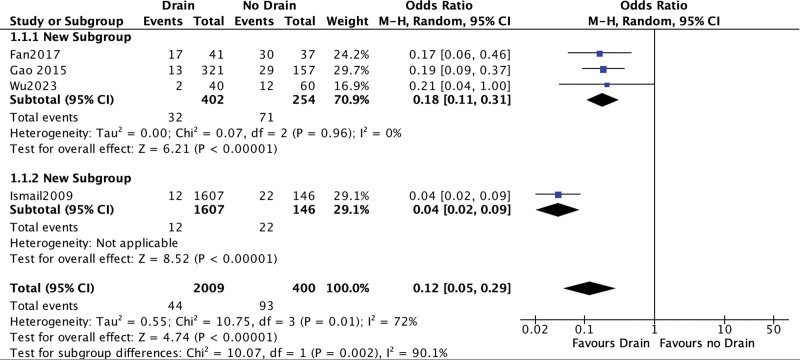
Postoperative seroma.

All included studies reported data on postoperative seroma.^[6–9]^ A statistically significant difference was noted favoring the patient group with prophylactic drainage (OR = 0.12; 95% CI [0.05, 0.29]; *P* < .001; 4 studies; *I*^2^ = 72%; Fig. 4). The source of heterogeneity was identified in the study by Ismail et al^[6]^ However, the subsequent subgroup with low heterogeneity still demonstrated a statistically significant effect favoring the prophylactic drainage patient group (OR = 0.18; 95% CI [0.11, 0.31]; *P* < .001; 3 studies; *I*^2^ = 0%). Noteworthy, based on GRADE judgement the level of evidence is low (Table S1, Supplemental Digital Content, http://links.lww.com/MD/L864).

### 3.4. Secondary outcomes

#### 3.4.1. Postoperative urinary retention.

**Figure 5.**
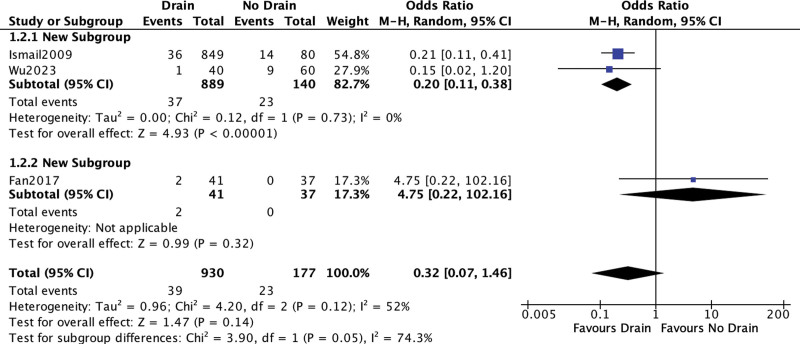
Urinary retention.

Data from 3 studies was pooled.^[6–8]^ No statistically significant difference was detected (OR = 0.32; 95% CI [0.07, 1.46]; *P* = .14; 3 studies; *I*^2^ = 52%; Fig. 5). Data pooling of Ismail et al^[6]^ and Wu et al^[8]^ demonstrated a significantly lower rate of urinary retention favoring the drain group, showing at the same time a high level of homogeneity (OR = 0.20; 95% CI [0.11, 0.38]; *P* < .001; 2 studies; *I*^2^ = 0%).

#### 3.4.2. Hospital length of stay.

**Figure 6.**
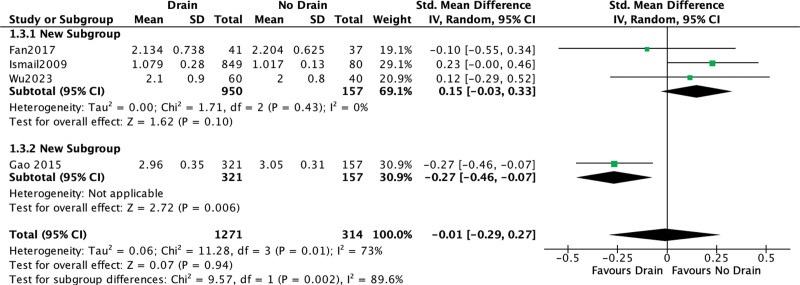
In-hospital stay.

This outcome was reported in all studies.^[6–9]^ No differences of the length of stay were detectable between the 2 study groups (SMD = −0.01; 95% CI [−0.29, 0.27]; *P* = .94; 4 studies; *I*^2^ = 73%; Fig. 6). The differences remained insignificant even after excluding the source of heterogeneity^[9]^ (SMD = 0.15; 95% CI [−0.03, 0.33], *P* = .1; 3 studies, *I*^2^ = 0%).

#### 3.4.3. Mesh infection.

All studies reported on mesh infection.^[6–9]^ The overall incidence was null.

#### 3.4.4. Recurrence.

**Figure 7.**
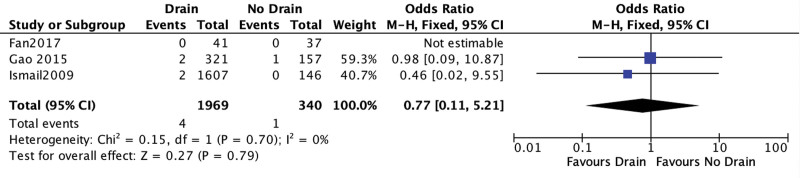
Recurrence.

Three studies reported on hernia recurrence. In the study of Fan et al^[7]^ no recurrence occurred, whereas Ismail et al^[6]^ noted 2 recurrent hernias at follow up both in the drainage group (OR = 0.77; 95% CI [0.11, 5.21]; *P* = .79; 3 studies; *I*^2^ = 0%; Fig. 7).

## 4. Discussion

TEP hernioplasty has proven itself at least as efficient and safe as the traditional open methods and has gained popularity among many laparoendoscopic hernia surgeons globally.^[15,16]^

A seroma refers to the accumulation of liquified fat, serum, and lymphatic fluid within a dead space. This is the case with the relatively extensively dissected myopectineal orifice following inguinal hernia repair. The development of seromas is frequently regarded as a complication following both laparoscopic and open hernia surgeries. It typically manifests shortly after the operation and in nearly all patients to varying degrees. The majority of seromas are absorbed over a span of weeks to months and remain asymptomatic, with only a small fraction of them necessitating their evacuation. As a result, seromas are seldom of clinical significance. This, often unremarkable, course is responsible for the lack of a universal definition of postoperative seromas in inguinal hernioplasty and hernia repair in general. Wu et al^[8]^ did define seroma as ‘the exudation and accumulation of fluid in the operation area’ but did not state how this was diagnosed. Ismail et al based their seroma definition solely on the clinical finding of a palpable fluid collection without a size limit whereas Fan et al^[7]^ defined seroma as ‘fluid collection with no Doppler signal at the preperitoneal space on ultrasound examination or clinically palpable, irreducible swelling after TEP hernioplasty with no cough impulse, a rather liberal definition that justifies the slightly higher seroma rates reported. No mesh infections occurred. This major complication is potentially linked to the presence of a drainage tube, as it is theoretically an entry point for contamination of the surgical site. All drainage tubes were removed at a relatively short time postoperatively (max. 48 hours) thus minimizing the chances of infectious complications. Little has been published referring to the correlation of drainage tubes and mesh infection in inguinal hernia surgery. In fact, Fan et al^[7]^ was the first one to produce grade 1 data through their RCT, showing no link between those 2 factors. A rather not so uncommon complication is postoperative urinary retention, with incidence being reported between 2% up to 30%. The presence of drainage tube did not seem to play a significant role regarding that matter in the meta-analysis. Visual analog scale (VAS) measurements of postoperative pain were conducted in all included studies. The relation of postoperative pain and presence of a drainage tube cannot be investigated within this meta-analysis as a remarkable inhomogeneity exists regarding the use of tacks for the fixation of the mesh, a notable confounding factor. Various approaches have been previously described to minimize seroma formation. Reddy et al^[17]^ focused on the direct inguinal hernia and advocated the eversion of the transversalis fascia at the hernia site and its fixation with tacks to eliminate the dead space and the subsequent seromas demonstrating favorable results. A further approach is the excision and/or ligation of the hernia sac.^[18]^ Insertion of drainage tubes to avoid seromas has been common practice for decades. Conversely just 4 studies that match our search criteria exist that control the hypothesis in TEP hernia repair. Our meta-analytic data show a clear benefit for patients in the drain group with regard to postoperative seroma formation. The source of heterogeneity with respect to the primary outcome was identified in the study of Ismail et al^[6]^ Pooling the remaining data after its exclusion show a persistently significant benefit for the drain group with maximal homogeneity (OR = 0.18; 95% CI [0.11, 0.31]; *P* < .001; 3 studies; *I*^2^ = 0%). The study by Gao et al^[9]^ was identified as source of heterogeneity with regard to the secondary outcome “in-hospital stay” that nevertheless, remained indifferent between the 2 study groups even after its exclusion (SMD = 0.15; 95% CI [−0.03, 0.33], *P* = .1; 3 studies, *I*^2^ = 0%). Subgroup analysis of the studies reporting on postoperative urinary retention showed a potential benefit for the drain group. That could be explained through reduced stimulus in the Retzius space. This assumption derives though from pooling of just 2 studies and should be cautiously interpreted.

Our meta-analysis has some limitations. Firstly, the number of studies that could be included was relatively low. A further limitation of our study is the fact all studies were conducted in Asia, hence, the included patients may not be representative of general patient population. Surgical expertise of the operating surgeons was variable, and thus a potential source of bias. The heterogeneity of the present meta-analysis with regard to inclusion of bilateral hernias may influence comparability of the presented results. Gao et al^[9]^ and Ismail et al^[6]^ included this patient subgroup, nevertheless without particular exploration of higher seroma formation risk in cases with bilateral hernia, and its potential benefit from a drain. This analysis would be of interest, as Hitman et al managed to demonstrate a tendency towards statistical significance with regard to postoperative seroma formation in patients with bilateral findings.^[19]^ Last but not least, the definition of postoperative seroma was not consistent throughout the studies, affecting the heterogeneity of the pooled data. However, this is to date the first meta-analysis of studies focusing exclusively on routine closed-suction drainage of the extraperitoneal space after TEP hernioplasty, supporting this prophylactic practice with regard to postoperative seroma formation.

## 5. Conclusion

The use of prophylactic closed-suction drainage of the extra-peritoneal space after TEP was associated with a reduced incidence of postoperative seroma, without demonstrating any additional morbidity. Due to the lack of high-quality trials and varying definitions of outcome measures for postoperative seroma, the results should be interpreted with caution.

## Author contributions

**Conceptualization:** Dimitrios Prassas, Stephan Oliver David, Wolfram Trudo Knoefel, Sascha Vaghiri.

**Data curation:** Dimitrios Prassas, Stephan Oliver David, Sascha Vaghiri.

**Formal analysis:** Dimitrios Prassas, Michael Zaczek, Stephan Oliver David, Sascha Vaghiri.

**Investigation:** Dimitrios Prassas, Sascha Vaghiri.

**Methodology:** Dimitrios Prassas, Michael Zaczek, Stephan Oliver David, Wolfram Trudo Knoefel, Sascha Vaghiri.

**Project administration:** Dimitrios Prassas, Wolfram Trudo Knoefel.

**Software:** Michael Zaczek, Stephan Oliver David, Wolfram Trudo Knoefel.

**Supervision:** Dimitrios Prassas, Wolfram Trudo Knoefel.

**Validation:** Sascha Vaghiri.

**Visualization:** Michael Zaczek, Stephan Oliver David.

**Writing – original draft:** Dimitrios Prassas, Wolfram Trudo Knoefel, Sascha Vaghiri.

**Writing – review & editing:** Dimitrios Prassas, Stephan Oliver David, Wolfram Trudo Knoefel.

## Supplementary Material


