# Supplementary material for: Routine closed-suction drainage reduces seromas following totally extraperitoneal (TEP) inguinal hernia repair: A meta-analysis
Source: Medicine (Baltimore). 2024 Mar 15;103(11):e37412. doi: 10.1097/MD.0000000000037412 (PMC10939558; doi:10.1097/MD.0000000000037412)
Supplement: Supplementary file 1 [file medi-103-e37412-s001.docx]

Table S1 GRADE assessment of significant outcomes

| Outcomes | No. of studies | **No. of included patients** | | OR [95 % CI] | **Quality assessment** | | | | | Quality |
| --- | --- | --- | --- | --- | --- | --- | --- | --- | --- | --- |
|  |  | Drain | No Drain |  | Risk of bias^a^ | Inconsistency | Indirectness | Imprecision | Publication bias |  |
| Postoperative Seroma | 4 [9,7,6,8] | 2009 | 400 | 0.12 [0.05,0.29] | Moderate | Serious | No indirectness | No imprecision | NA | Low |
| NA: not applicable, OR: odds ratio ^a^ Risk of bias assessed using the ROBINS-I and RoB 2 tools. | | | | | | | | | | |
